# Placental site trophoblastic tumor and choriocarcinoma: an unusual presentation

**DOI:** 10.1186/s13104-015-1693-8

**Published:** 2015-11-23

**Authors:** Abdulrahim Gari

**Affiliations:** Department Of Obstetrics and Gynecology, College of Medicine, Umm Al-Qura University, PO Box : 6707, Makkah, Saudi Arabia

**Keywords:** Adjuvant chemotherapy, β-hCG, Choriocarcinoma, Hysterectomy, Pathology

## Abstract

**Background:**

Mixed trophoblastic tumor composed of choriocarcinoma and placental site trophoblastic tumor was diagnosed on histopathology slides review, is a very rare mixed tumor and cured after adjuvant chemotherapy.

**Case presentation:**

Twenty-nine years old, Para 2 female presented with 4 months history of irregular vaginal bleeding after an uncomplicated vaginal delivery. Abdominal examination showed 14 weeks uterus with β-hCG level of 14,889 mIU/ml. Slides review confirmed the diagnosis of placental site trophoblastic tumor (PSTT). Patient underwent total abdominal hysterectomy and resection of anterior vaginal wall mass. Post operative (48 h) β-hCG level was 6016 mIU/ml. Final pathology showed mixed trophoblastic tumor composed of choriocarcinoma (CC) and PSTT. Adjuvant chemotherapy started and continued for three cycles after achieving normal β-hCG.

**Conclusion:**

PSTT is a rare disease and potentially curable. Differential diagnosis in women presented with vaginal bleeding and a uterine mass in the post partum period must include gestational trophoblastic disease.

## Background

A rare form of gestational trophoblastic disease (GTD) is placental site trophoblastic tumor (PSTT). It arises from invasive intermediate gestational trophoblasts and constitutes 1–2 % of all gestational trophoblastic neoplasia (GTN) [1]. It was considered a benign lesion before 1981 when Scully and Young recognized that it was potentially malignant [2]. Approximately 200 cases have been reported in the literature [3]. The incidence of GTN varies in different regions from 0.6 to 1.1/1000 pregnancies in Europe and North America, while 2/1000 pregnancies in Japan and 1/160 pregnancies in India and Middle East [4].

Here we present a rare case of mixed trophoblastic tumor consisting of choriocarcinoma (CC) and PSTT. It was diagnosed after term normal delivery and managed with surgery followed by adjuvant chemotherapy.

## The case presentation

A 29 years old female, Para 2 presented with irregular vaginal bleeding started 4 months after an uncomplicated vaginal delivery of a term male infant. Her first delivery was also normal without any antenatal or postnatal complication. Otherwise her history was unremarkable. In referring hospital she was diagnosed as a case of retained product of conception on ultrasound. Serum beta human chorionic gonadotrophin (β-hCG) level was 4253 mIU/ml. Dilatation and curettage (D&C) done. Final pathology showed PSTT.

She was referred on January 28, 2013 for assessment and management to our Oncology centre at King Abdullah Medical City, Makkah, Saudi Arabia. On examination, a thin lean lady was markedly pale with tachycardia. Abdominal examination showed 14 week palpable uterus. Local examination showed mild bleeding, healthy external genitalia, and healthy cervix/vagina. At that time β-HCG was 14,889 mIU/ml. Her Hemoglobin (Hb) was 7.3 and she received four units of packed red blood cells (RBCs). Review of slides showed a neoplasm composed of proliferation of polyhedral mononuclear intermediate trophoblasts with extensive deposition of fibrinoid material confirming the diagnosis of PSTT.

Metastatic workup with CT scan of chest, abdomen, and pelvis done on February 2, 2013 showed innumerable pulmonary nodules scattered in both lungs, largest 8.4 mm in right middle lobe. Seven centimeter (cm) heterogenous mass filled the endometrial canal and extending to the level of cervix. The parametria were slightly hazy but no definite involvement was found with prominent both adenexa. No evidence of ascites nor lymphadenopathy (pelvic, abdominal, inguinal). All other structures were within normal limits.

Patient offered surgery (total abdominal hysterectomy) followed by chemotherapy but she refused treatment and left the hospital against medical advice. Four weeks later, she presented to emergency department with heavy vaginal bleeding. Hemoglobin was 6.6 mg/dl and received four units of packed RBCs. Abdominal examination showed 22 week sized uterus. A newly developed anterior vaginal wall mass of 4 × 4 cm was found. CT scan repeated CAP showed interval worsening of pulmonary nodules and enlarged uterus with anterior vaginal mass.

**Fig. 1:**
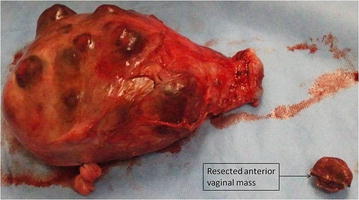
Uterus with multiple areas of impending tumor perforation with resected anterior vaginal mass

**Fig. 2:**
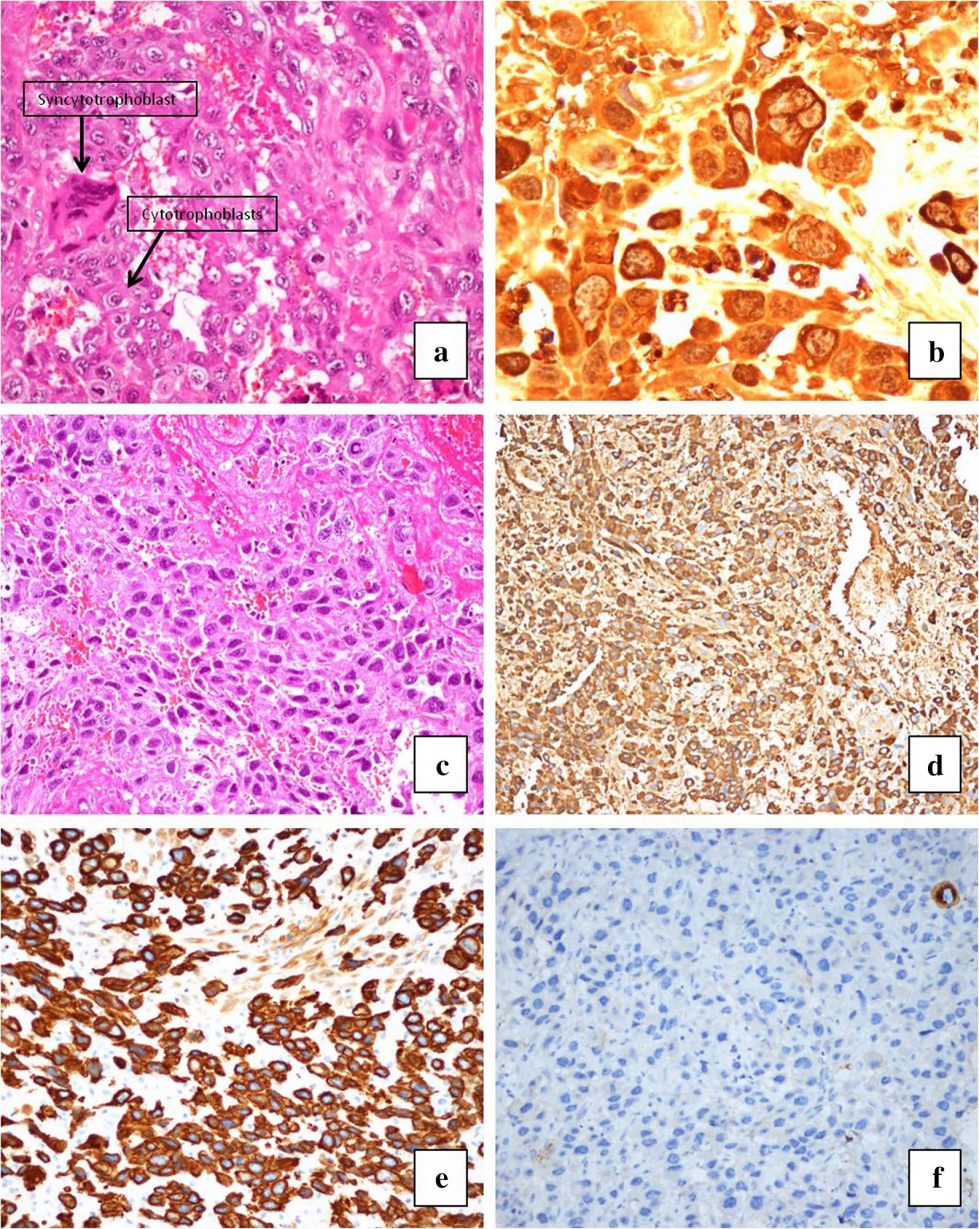
**a** Hematoxylin and eosin (H & E) stain showing dimorphic picture of syncythiotrophoblast and cytotrophoblast in Choriocarcinoma; **b** β-Human Chorionic Gonadotrophin immunohistochemical stain showing positive staining in Choriocarcinoma componants; **c** H & E Stain showing sheets of monomorphic intermediate trophoblasts and fibrinoid material in placental site trophoblastic tumor; **d** Immunohistochemistry (IHC): hPL immunostain showing positive staining in intermediate trophoblasts of placental site trophoblastic tumor; **e** IHC: Inhibin immunostain showing positive staining in intermediate trophoblasts of placental site trophoblastic tumor; **f** IHC: β-HCG immunostain showing negative staining in intermediate trophoblasts of placental site trophoblastic tumor

Patient agreed for surgery, underwent total abdominal hysterectomy and excision of anterior vaginal mass on March 3, 2013. Intra operatively uterus was found to be enlarged (20 weeks sized) with multiple areas of impending tumor perforation. Both tubes and ovaries were normal with no ascities or any intra-abdominal bleeding. Post-operative course was unremarkable. Repeated β-hCG (48 h post-op) was 6016 mIU/ml. The microscopic examination of uterus showed mixed trophoblastic tumor composed of chroriocarcinma (CC) and PSTT. Invasion of myometrium, and lymph-vascular invasion were identified. The CC component showed typical dimorphic picture consisting of syncytiotrophoblasts and cytotrophoblasts. Immunohistochemical staining for β-hCG revealed positive staining in this component. The PSTT which constitute about 20 % of tumor showed sheets of monomorphic intermediate trophoblasts and fibrinoid material. These cells were strongly positive for human placental lactogen (hPL), and inhibin, and negative for β-hCG, and P-63. Ki-67 proliferation index was high in CC component. (Figs. 1, 2).

Three weeks post surgery, combination chemotherapy EMA/CO protocol was started and continued 3 cycle after achieving normal β-hCG (quantitative β-hCG <4 mIU/ml). CT scan of the chest showed complete resolution of pulmonary metastasis. Currently she is in remission and under monthly follow up.

## Discussion

PSTT is rare disease among the other variants of GTN, i.e., choriocarcinoma (CC) the most common and epithelioid trophoblastic tumor (ETT) that is also relatively rare [5]. It is an extravillous infiltrating tumor, which can invade the myometrium and arises from intermediate trophoblasts [6].

PSTT produces significantly lower serum levels of hPL and serum β-hCG than does CC. On the other hand some cases of PSTT with high serum β-hCG have been also reported. PSTT may complicate or follow a normal term pregnancy, abortion or molar pregnancy. It can present months or years after a term gestation with a variety of clinical features like; irregular vaginal bleeding, amenorrhea, nephrotic syndrome, and rarely, polycythemia or virilization. It is usually confined to the uterus at the time of diagnosis, but on later stage it has high risk of metastasis to the lungs and vagina [1]. It is less chemo sensitive unlike other forms of GTD and produces a lesser amount of β-hCG. As a result, surgery is the core treatment but adjuvant chemotherapy is increasingly being offered in cases of metastasis and large tumor volume [2, 7]. GTD has two established risk factors, i.e., reproductive age (<20 and >40 years) and previous molar pregnancy. One case of Epithelioid Trophoblastic Tumour (ETT) and CC has been reported in Australia in 2013 [8]. Our case has no such risk factors, and up to our knowledge no such a case of PSTT and CC has been reported in literature yet.

## Conclusion

PSTT is a rare disease and potentially curable. Differential diagnosis in women presented with vaginal bleeding and a uterine mass in the post partum period must include GTD. Gold standards for diagnostic evaluation of GTD is β-hCG and tissue diagnosis. For patients with disease localized to the uterus, surgery remains the mainstay of therapy in the first instance, in contrast to other GTDs, because of it is low chemosensitivity. EMA/CO chemotherapy should be considered for metastatic cases.

## Consent

Written informed consent was obtained from the patient for publication of this Case report and any accompanying images. A copy of the written consent is available for review by the Editor of this journal.

## References

[1] Cole ME, Broaddus R, Thaker P, Landen C, Freedman RS (2008). Placental-site trophoblastic tumors: a case of resistant pulmonary metastasis. Nat Clin Pract Oncol.

[2] Piura B (2006). Placental site trophoblastic tumor e a challenging rare entity. Eur J Gynaecol Oncol.

[3] Colecchi C, Partemi S, Minelli N, Cascini F, Rossi R, Fulcheri E, Pascali VL, Oliva A (2011). Placental site trophoblastic tumor with lung metastases as cause of death in a young patient: a case report. Placenta.

[4] Sudha CP, Sahana M (2014). Chemoresistant gestational trophoblastic neoplasia: a case report. J Clin Diagn Res.

[5] Ajithkumar TV, Abraham EK, Rejnishkumar R, Minimole AL (2003). Placental site trophoblastic tumor. Obstet Gynecol Surv.

[6] Nupur G, Sharma JB, Suneeta M, Divya T, Lalit K, Manu K (2010). Placental site trophoblastic tumor of the uterus: a mistaken diagnosis. JK Sci.

[7] Parul Agarwal N, Kriplani A, Vijayaraghavan M (2002). Placental site trophoblastic tumour. J Postgrad Med.

[8] Luk WY, Friedlander M (2013). A fibroid or cancer? A rare case of mixed choriocarcinoma and epithelioid trophoblastic tumour. Case Rep Obstet Gynecol.

